# Partial response of pulmonary adenocarcinoma with symptomatic brain metastasis to nivolumab plus high-dose oral corticosteroid: a case report

**DOI:** 10.1186/s13256-017-1334-z

**Published:** 2017-07-06

**Authors:** Hélène Pluchart, Julian Pinsolle, Julien Cohen, Gilbert R. Ferretti, Pierrick Bedouch, Matteo Giaj Levra, Anne-Claire Toffart, Denis Moro-Sibilot

**Affiliations:** 1 Pôle pharmacie, CHU Grenoble Alpes, CS 10217, 38043 Grenoble, France; 2 Clinique Universitaire de Pneumologie, Pôle Thorax et Vaisseaux, CHU Grenoble Alpes, CS 10217, 38043 Grenoble, France; 3 Pôle radiologie et imagerie médicale, CHU Grenoble Alpes, CS 10217, 38043 Grenoble, France; 4 Université Grenoble Alpes/CNRS, ThEMAS TIMC UMR 5525, Grenoble, F-38041 France; 5 Institut pour l’Avancée des Biosciences, CRI UGA/Inserm U 1209/CNRS UMR 5309, Grenoble, France

**Keywords:** Case report, Pulmonary adenocarcinoma, Brain metastasis-related symptoms, Immunotherapy, Immunomodulating drugs, Nivolumab, Corticosteroids

## Abstract

**Background:**

Nivolumab, a monoclonal antibody targeting the programmed death-1 receptor, is indicated in locally advanced or metastatic non-small cell lung cancer, with progression after platinum-based chemotherapy. Up-to-now, few data are available concerning brain activity of this treatment and concomitant use of corticosteroids.

**Case presentation:**

A 64-year-old caucasian man with a pulmonary adenocarcinoma associated with brain metastases received four courses of nivolumab in concomitance with a high dose of corticosteroids for his neurologic symptoms. He experienced a partial response in his brain and chest with an improvement in his general condition.

Nivolumab was effective in shrinking symptomatic brain metastases, and metastases at other sites, in a patient with non-small cell lung cancer and first-line chemotherapy failure. The effect of nivolumab was obtained despite concomitant high-dose corticosteroid therapy. Combined nivolumab and high-dose corticosteroid therapy did not induce unexpected adverse events.

**Conclusion:**

Nivolumab and concomitant high-dose corticosteroid therapy was found to be efficient and well tolerated.

## Background

The standard of care for non-small cell lung cancer (NSCLC) has changed with the introduction of immune checkpoint modulators such as nivolumab, a monoclonal antibody which binds to the programmed death-1 (PD-1) receptor expressed on T cells. In phase III trials, nivolumab improved both overall survival (OS) and progression-free survival (PFS) [1], or only PFS [2] compared with docetaxel, in locally advanced or metastatic NSCLC with progression after first-line chemotherapy. In these trials, patients with unstable or untreated brain metastases were excluded and the highest permitted corticosteroid dosage was 10 mg daily prednisone (or equivalent) within the last 2 weeks.

We describe a patient with metastatic pulmonary adenocarcinoma who received nivolumab with oral corticosteroid therapy for symptomatic brain metastasis.

## Case presentation

In June 2014, a 64-year-old caucasian man with a 40 pack-year tobacco smoking history was diagnosed with stage IV, *KRAS* mutated (glycine substitution at the codon 12 in exon 2), adenocarcinoma of the lung. He had detectable lung and mediastinal lymph node metastases but no brain metastasis. Between August 2014 and December 2015, he successively received cisplatin and pemetrexed, docetaxel, erlotinib, then gemcitabine. In December 2015, thoracic and mediastinal progression, as well as new vertebral, pleural, and adrenal metastases were diagnosed. He had no neurological symptoms and, consequently, brain imaging was not performed. In January 2016, he started nivolumab 3 mg/kg every 15 days.

**Fig. 1:**
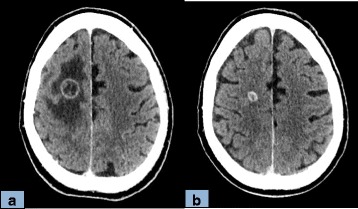
Brain computed tomography scan comparison after the first (**a**) and the fourth (**b**) nivolumab infusion. Computed tomography scan, view through the corona radiata 6 days after the first nivolumab injection (**a**) and after four courses of nivolumab plus oral corticosteroid therapy (**b**). The large frontal rim-enhanced metastasis decreased substantially in size, from 21 to 9 mm, and the surrounding edema resolved completely

**Fig. 2:**
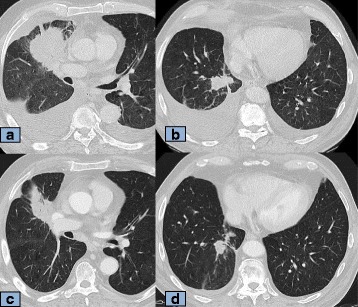
Thoracic computed tomography scan comparison after the first and the fourth nivolumab infusion. Computed tomography scan, axial views through the middle and lower lung lobes before the first nivolumab injection (**a** and **b**) and after four courses of nivolumab and oral corticosteroid therapy (**c** and **d**). Both the primary tumor in the middle lobe and the metastasis in the lower lobe of the right lung shrank substantially (from 80 to 60 mm and from 23 to 16 mm, respectively). The amount of fluid in the two pleural cavities also decreased

Three days after the first infusion, left hemiparesis developed. A brain computed tomography (CT) scan showed at least four brain metastases including a right frontal lesion with significant perilesional edema consistent with the neurological symptoms (Fig. 1). He received 80 mg of oral prednisolone per day. The hemiparesis resolved within 10 days. Nivolumab dosage and time of administration were left unchanged. After 1 month, the corticosteroid dose was lowered. Imaging studies performed after four courses of nivolumab showed almost complete disappearance of the pleural and adrenal metastases, together with substantial shrinkage of the other lesions: for example, from 21 to 9 mm for the right frontal brain metastasis (Fig. 1) or from 80 to 60 and from 23 to 16 mm for the primary lung tumor in the middle lobe and the metastasis in the lower lobe of his right lung, respectively (Fig. 2). The treatment was well tolerated, although hyperthyroidism developed.

Four months after nivolumab start, the drug remained radiologically and clinically effective: Eastern Cooperative Oncology Group (ECOG) Performance Status of 0.

**Fig. 3:**
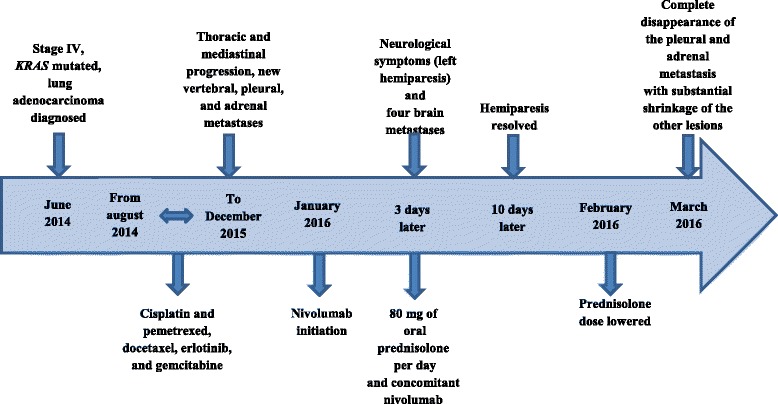
Timeline summarizing clinical history and therapeutic interventions

The timeline summarizes clinical history and therapeutic interventions (Fig. 3).

## Discussion

Nivolumab is approved in Europe and United States of America (USA) for the second-line treatment of NSCLC [3, 4]. Corticosteroids have immunosuppressive effects and were therefore excluded during the pivotal trials of nivolumab. However, during nivolumab therapy, symptoms requiring corticosteroid therapy may appear. In theory, concomitant corticosteroid therapy might decrease the effectiveness of nivolumab.

In a pooled analysis of data from the KEYNOTE-001 trial, in which 550 patients with advanced NSCLC took pembrolizumab, another monoclonal antibody targeting PD-1, some patients received corticosteroid therapy to treat immune-related adverse events [5]. PFS was not significantly different between these patients and those who did not take corticosteroids. However, pembrolizumab was discontinued during corticosteroid therapy. CheckMate 017 and Checkmate 043 evaluated the efficiency and safety of nivolumab in advanced squamous NSCLC. The pooled analysis of the data from these two trials included 248 patients given nivolumab. Immune-related adverse events requiring corticosteroid therapy were uncommon and usually low-grade events that resolved within 6 weeks in most cases. In both trials, nivolumab was also discontinued during corticosteroid therapy [6].

Few data are available about immunotherapy efficiency on primary or metastatic brain tumors [7, 8]. Ipilimumab has been found effective in melanoma with asymptomatic brain metastases [9]. Of 12 patients with brain metastases from NSCLC treated with nivolumab, 7 (58%) required discontinuation of the drug because of exacerbation of their neurological symptoms [10]. Data from available trials suggest that immunotherapy may have similar effects on brain and extracerebral metastases [11]. NSCLC often causes brain metastases, for which no specific treatment is available. Nivolumab Italian Expanded Access Program (EAP) is a program developed in order to include patients with asymptomatic brain metastases from squamous NSCLC in clinical trials involving nivolumab [12]. This approach aims to evaluate the efficiency and safety of nivolumab use in this subpopulation given the scarce available data. Among 372 patients, 38 patients with asymptomatic brain metastases received nivolumab. Only one patient discontinued nivolumab because of an adverse event. There was 1 complete response, 6 partial responses, and 11 stable diseases. Median PFS was 5.5 months and OS was 6.5 months. Since many patients are diagnosed stage IV NSCLC with brain metastases, the evaluation of these outcomes is of major clinical significance. These data toward nivolumab activity in brain metastases of squamous NSCLC, although preliminary, are encouraging the evaluation of nivolumab efficiency and safety in this cohort of real-life patients, who are excluded from clinical trials. This is why further investigations of immunotherapy in this subpopulation must be pursued [13, 14].

In our patient, despite the symptomatic brain metastasis and high-dose of oral corticosteroid therapy, nivolumab was highly effective on tumors at all sites and induced no unexpected severe toxicity. Thus, the high dose of corticosteroid therapy did not seem to decrease the effectiveness of nivolumab.

## Conclusion

This original case report suggests that nivolumab may be effective and safe in patients with brain metastases from NSCLC who are taking concomitant high-dose corticosteroid therapy.

## Abbreviations

CT: Computed tomography
NSCLC: Non-small cell lung cancer
OS: Overall survival
PD-1: Programmed death-1
PFS: Progression-free survival
